# Effect of temperature on structural, dynamical, and electronic properties of Sc_2_Te_3_ from first-principles calculations†

**DOI:** 10.1039/d2ra05720d

**Published:** 2022-11-15

**Authors:** Getasew Mulualem Zewdie, Tekalign Terfa Debela, Georgies Alene Asres

**Affiliations:** College of Mechanical and Industrial Engineering, Institute of Technology, Dire-Dawa University Dire-Dawa Ethiopia getasewmzewdie@hotmail.com; Institute for Application of Advanced Materials, Jeonju University Chonju Chonbuk 55069 Republic of Korea; Center for Materials Engineering, Addis Ababa Institute of Technology, School of Multi-disciplinary Engineering Addis Ababa 1000 Ethiopia

## Abstract

The compounds Sc_2_Te_3_ and Sb_2_Te_3_ have the same crystal structure. Ge–Sb–Te alloys are also the most common prototype phase change memory (PCM) compounds in the GeTe–Sb_2_Te_3_ pseudo-binary combination. Recently, alloying Sc atoms into Sb_2_Te_3_ has enabled sub-nanosecond switching in large conventional phase-change random access memory (PCRAM) devices. However, prior study on the electronic structure and dynamic properties of the Sc_2_Te_3_ system is very limited. In this work, we investigate the effect of temperature on the structural, dynamic, and electronic properties of the Sc_2_Te_3_ compound through *ab initio* molecular dynamics simulations. We show that the distorted-octahedral clusters are connected by four-fold rings even at higher temperatures. Moreover, our results clearly illustrate a liquid-to-glass transition temperature, which is between approximately 773 K and 950 K. The effect of temperature changes on the electronic properties of the system manifests as a metal-to-semiconductor transition. The band gap obtained using the mBJLDA functional is twice the value obtained using the PBE functional. Our studies provide useful insight into the local structure and dynamic and electronic properties of the Sc_2_Te_3_ system at the atomic level. We hope that this work could stimulate more theoretical work on the development of cache-type phase-change memory and broaden its application in the field of PCM.

## Abstract 2

We employed AIMD simulations to investigate the effect of temperature on the electronic structure and dynamical properties of Sc_2_Te_3_ material, which also manifests as a metal-to-semiconductor transition upon heating.

## Graphical abstract

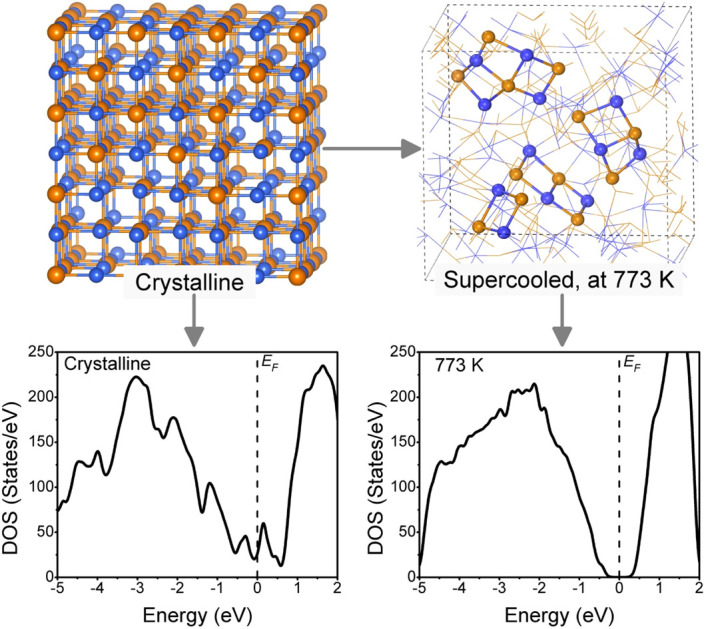

## 1. Introduction

Chalcogenide phase-change materials (PCMs) are widely used for large-capacity rewritable optical and electronic data storage devices that take advantage of the reversible and fast switching between the amorphous and crystalline phases.^1,2^ Among chalcogenide alloys, Ge–Sb–Te is a representative PCM along with the GeTe–Sb_2_Te_3_ pseudo-binary combination, which has been used in phase-change random access memory (PCRAM) technology.^3–5^ However, due to the long crystallization time, the SET (crystallization) speed is one of the major challenges for the full application of PCMs. Significant efforts have been made to increase the insufficient operating speed of phase-change memories.^6–11^ The alloying of Sc atoms in Sb_2_Te_3_ opens up the possibility of using phase-change devices to build a universal memory. This new material enables sub-nanosecond memory writing without preprogramming in a large conventional PCRAM device.^12^ This ultrafast crystallization is due to the reduced stochasticity of nucleation through geometrically matched Sc_2_Te_3_ and Sb_2_Te_3_, and robust Sc–Te chemical bonds that stabilize the crystal precursors in the amorphous state.^12,13^

The compound Sc_2_Te_3_ has a cubic rocksalt structure with 1/3 vacancies on the cationic sub-lattice of the crystalline phase, which corresponds to that of cubic rocksalt Sb_2_Te_3_. As an important prototypical PCM, Sb_2_Te_3_ has been the subject of much theoretical and experimental research.^14–17^ On the other hand, there are few reports on the liquid and amorphous states of Sc_2_Te_3_ according to theoretical studies. Previous work on the Sc_2_Te_3_ system has only reported the structural and chemical bonding characteristics in the amorphous state.^13^ However, knowledge about the effect of temperature on the electronic structure and dynamic properties of the Sc_2_Te_3_ compound is still insufficient and it needs to be further investigated.

In this work, we study the effect of temperature on the structural, dynamic, and electronic properties of the Sc_2_Te_3_ compound through *ab initio* molecular dynamics simulations (AIMD). Pair correlation function (PCF), coordination number (CN), angular distribution functions (ADF), and primitive ring (PR) analysis are used to explore the structural characteristics of the Sc_2_Te_3_ system. The mean-squared displacement (MSD) and Wendt–Abraham (WA) parameters are calculated to study the dynamic properties of the Sc_2_Te_3_ system. The electronic properties of the Sc_2_Te_3_ system are analyzed using the density of states (DOS) and Bader charge analyses. Moreover, the changes in the dynamic properties of Sc_2_Te_3_ from the liquid-to-glassy state are also explored.

## 2. Computational details

All models of the Sc_2_Te_3_ system at different temperatures are modeled through AIMD simulations based on density functional theory (DFT). The second-generation Car–Parrinello scheme is used as implemented in the Quickstep code of the CP2K package.^18^ The code uses a mixed scheme of Gaussian-type basis sets and plane waves. The Kohn–Sham orbitals are expanded in basis sets with triple-zeta plus polarization quality. To expand the charge density, plane waves with a cutoff of 300 Ry are used. The Goedecker–Teter–Hutter (GTH) pseudopotentials^19^ and gradient-corrected Perdew–Burke–Ernzerhof (PBE) functional^20^ are used. The Brillouin zone is sampled at the *Γ* point of the supercell. A stochastic Langevin thermostat^21,22^ controls the temperature in the canonical NVT (constant number of particles, volume, and temperature) ensemble. The time step for the simulations is 2 femtoseconds.

The initial configuration is modeled using a 3 × 3 × 3 supercell of the cubic rocksalt structure (72 Sc and 120 Te atoms). The supercell is thermally equilibrated at a high temperature much higher than its melting temperature to eliminate the memory effect of the initial configuration. The system is then quenched to the melting temperature and equilibrated there for 30 ps. Next, the liquid model is quenched down to 300 K in 50 ps and equilibrated at 300 K for 30 ps. In the quenching process, the internal stress is precisely controlled by adjusting the box size. The theoretical density of 0.0313 atom per Å^3^ is used for the Sc_2_Te_3_ system at 300 K.^13^ Finally, the system is run for 7500 MD steps at each temperature for statistical analysis. The last 7000 trajectories are used for structural characterization and dynamic analysis. At each temperature, the volume of the system is adjusted by changing the size of the simulation box to ensure that the internal pressure of the system is close to zero. The primitive ring distribution analysis is performed using the RINGS code.^23^

The electronic properties are performed with the VASP (Vienna *Ab initio* Simulation Package) code.^24,25^ A plane wave kinetic energy cutoff of 450 eV is used under the projector augmented wave (PAW) method.^26^ Since the standard semilocal exchange-correlation functional usually underestimates the band gap, the modified Becke–Johnson exchange potential in combination with LDA-correlation (mBJLDA)^27^ is employed to calculate the DOS. Moreover, the experimental lattice parameters are used for DOS calculations of the crystal phases of the Sc_2_Te_3_ system.

## 3. Results and discussion

### 3.1. Local structural order

**Fig. 1:**
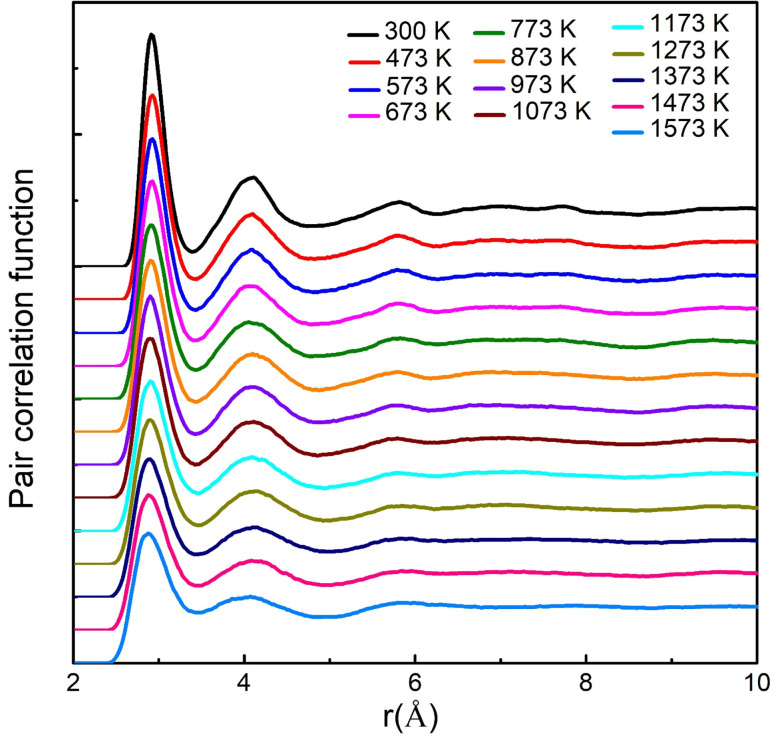
The total pair-correlation functions (PCF) of the Sc_2_Te_3_ system at different temperatures. Note that, for clarity, each curve has been vertically displaced from the curve below.

**Fig. 2:**
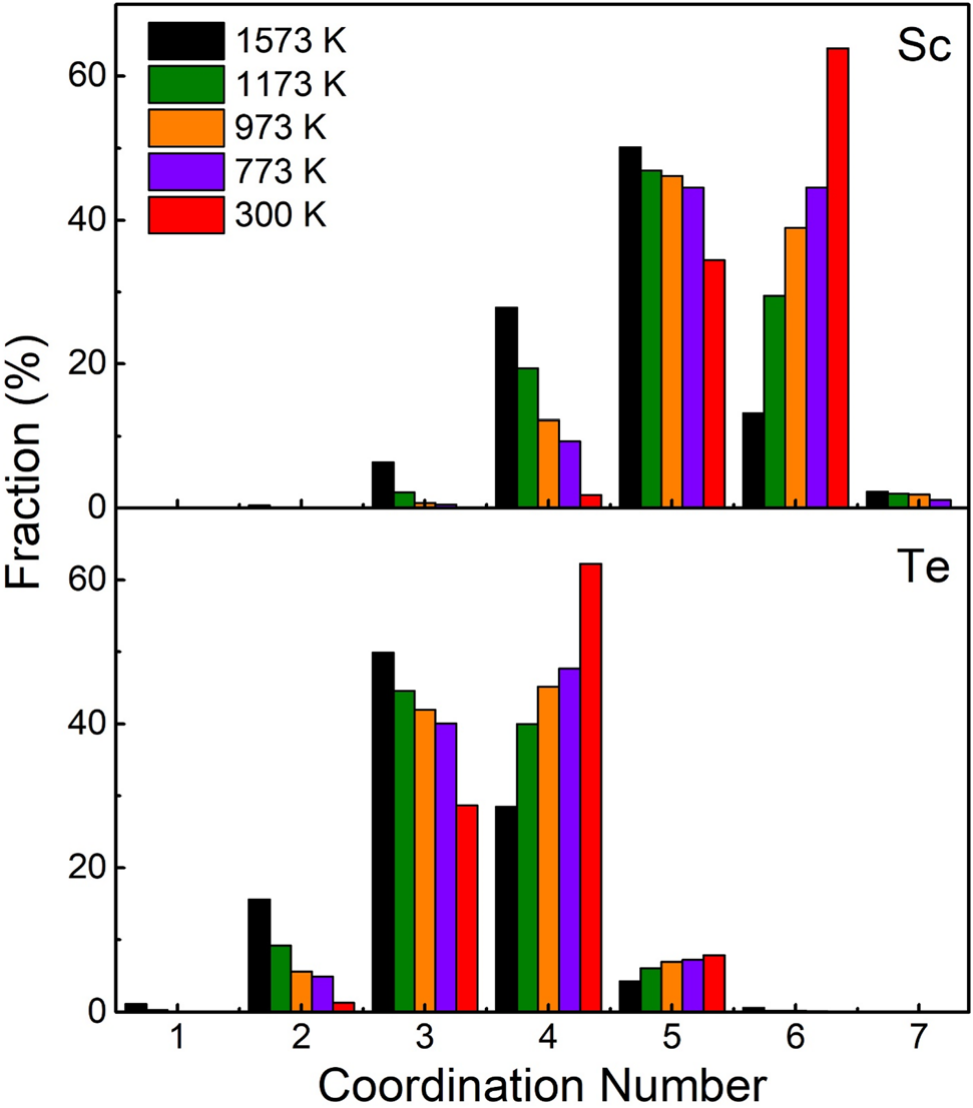
The coordination number (CN) distributions for the Sc- and Te-centered atoms in the Sc_2_Te_3_ system at different temperatures.

The PCF can be defined as the normalized possibility (relative to the uniform probability) of finding an atom at a distance *r* from the central atom, and has been widely used to characterize the structure of liquids and glassy states. The total PCF of Sc_2_Te_3_ as a function of temperature is shown in Fig. 1. The first peaks in the total PCF become stronger as the temperature decreases. On the one hand, the first valley becomes deeper and the second and third peaks become more pronounced, indicating that the system is more ordered as temperature decreases. The first peak of the total PCF at temperatures below 573 K is located at about 2.93 Å. This peak decreases slightly with increasing temperature, but the difference is insignificant. This value is in good agreement with the experimental interatomic distances (=2.91 Å) in the cubic Sc_2_Te_3_ structure.^28^ As the temperature increases, the atoms move more vigorously, and multiple snapshots need to be averaged to determine CNs. Fig. 2 shows the average total CNs of the last 7000 MD trajectories at each temperature. Moreover, since CN depends on the cutoff value, a uniform cutoff of 3.3 Å is used to determine CNs. This cutoff value is close to the first minimum in the total PCF. The figure clearly shows that Sc-centered atoms are dominated by six- and five-coordination, while Te-centered atoms are mainly four- and three-coordinated. As the temperature increases, the number of high-coordination polyhedra gradually decreases, while the number of low-coordination polyhedra begins to increase. These results indicate that the high-coordination Sc-cluster is gradually formed in the amorphous state of the Sc_2_Te_3_ system. The A–B partial CNs in the Sc_2_Te_3_ at different temperatures (A is the central atom and B is the coordination atom) are displayed in Fig. S1.† The CNs of Sc–Te and Te–Sc increase significantly with decreasing temperature, indicating tighter packing between the Sc and Te atoms. On the one hand, a small number of homopolar bonds (such as Sc–Sc, and Te–Te) exist at high temperatures. However, such homopolar bonds are completely absent at low temperatures. In the amorphous and supercooled states of Sc_2_Te_3_, the average CNs of Sc and Te atoms are ∼5.64 and ∼3.76, respectively, which can be compared with the ideal octahedral and tetrahedral coordination, respectively. The average CNs of the different pairs of atoms are shown in Table S1.†

**Fig. 3:**
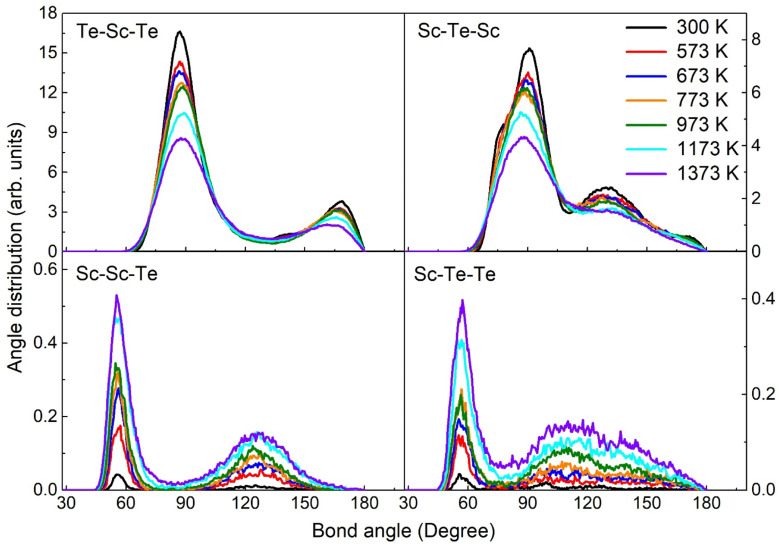
The calculated partial angular distribution function (ADF) of the Sc_2_Te_3_ system at different temperatures.

To further explore the effect of temperatures on the local-bonding environment in the Sc_2_Te_3_ system, we also calculate the ADF. Fig. 3 shows the partial ADF at different temperatures. The figure shows that the most prominent peaks are located in Te–Sc–Te and Sc–Te–Sc at lower temperatures, namely at 90° and 170°, which are the characteristic peaks of defective octahedral geometry. On the one hand, the shoulder and small peaks of Sc–Te–Sc around 70° and 130° also emerge at lower temperatures. This is mainly due to the non-octahedral bonding environments in the rhombohedral crystal phase of Sc_2_Te_3_.^13^ So far, the peaks at bond angles of 90° and 170° become sharper with the decrease in temperature, revealing that the octahedral configuration becomes more ordered. In contrast, the main peaks of Sc–Sc–Te and Sc–Te–Te are around 57°, whereas the bond angle distributions of Sc–Sc–Sc and Te–Te–Te are not found. This result indicates the strong interaction between the Sc and Te atoms in the Sc_2_Te_3_ system. Remarkably, homopolar bonds are almost negligible even at higher temperatures.

**Fig. 4:**
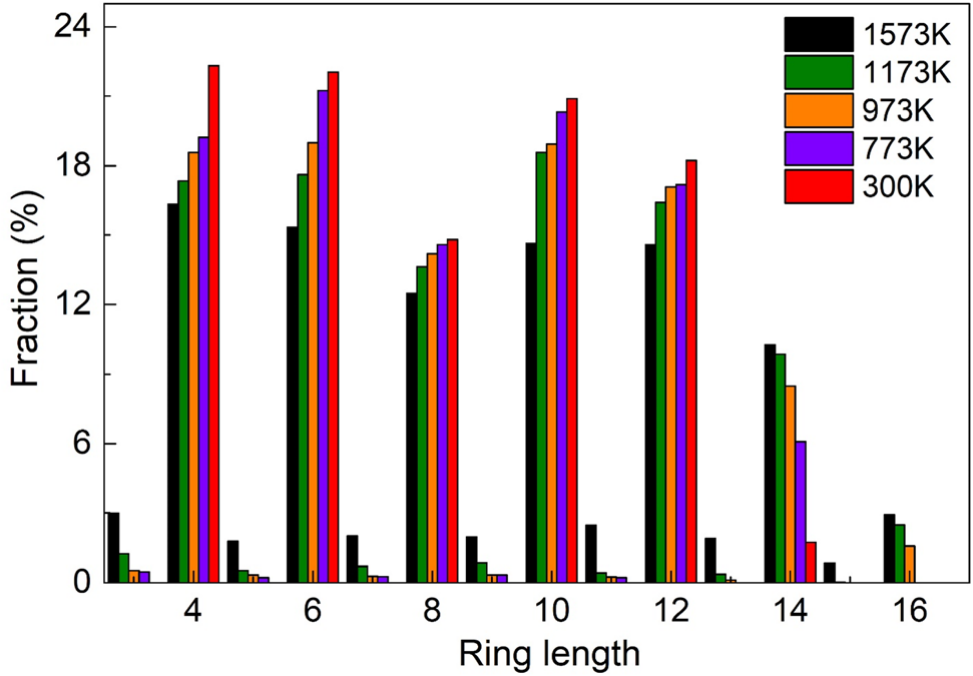
The distribution of medium-range-order (MRO) in the Sc_2_Te_3_ system at different temperatures.

To understand the medium-range order (MRO) in the Sc_2_Te_3_ system in more detail, we show the PR distributions. This technique can provide insight into the connectivity of the topological network of disordered materials. Unlike the ideal rocksalt lattice, which is dominated by four-membered rings, more complex rings could appear in the disordered states. The PR distribution of the Sc_2_Te_3_ system as a function of temperature is shown in Fig. 4. We use the same cutoff distance as the CN distribution. Similar to the crystalline phases of the Sc_2_Te_3_ structure (Fig. S2†), even-membered rings are the main structural motifs in the amorphous and liquid states. At lower temperatures, four-fold and six-fold rings account for a large proportion. The absence of odd-membered rings is due to the absence of Sc–Sc and Te–Te bonds. The disappearance of homopolar bonds in the Sc_2_Te_3_ system is due to the favored Sc–Te interaction. As a result, the four-fold rings in the Sc_2_Te_3_ system are completely dominated by ABAB squares (A = Sc, B = Te). Fig. S3† shows typical ABAB squares found in our simulations. The four-fold rings are the basic structural elements for fast phase transition in PCMs.^29–32^ In this regard, the crystal-like amorphous structure plays a vital role in promoting nucleation and reducing the structural arrangement, thereby ensuring fast crystallization speed, such as in Sc-doped Sb_2_Te_3_ (ref. 12) and Bi-doped Sb_2_Te_3_ (ref. 33) PCMs. Highly coordinated octahedra can provide more structural sites for the formation of four-fold rings; thus the less distorted octahedral motifs in the Sc_2_Te_3_ system are more conducive to the formation of these four-fold rings. This finding differs from the most commonly used PCMs, as most supercooled PCMs have large homopolar bonds.^34,35^

### 3.2. Dynamic properties

Diffusion is the process caused by the movement of atoms from an area of higher concentration to one of lower concentration across the host material. MSD is a widely used method that contains information about atomic diffusion.^36^ Mathematically MSD as a function of time is defined as follows:1
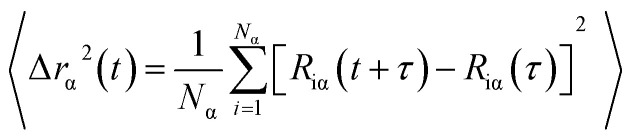
where *r*_α_^2^ is mean square displacement, *α* is the atom type and *t* is time. The angular brackets < > indicate an average over initial (at time *τ* = 0) velocities and positions of the molecules. The number of α atoms is denoted by *N*_α_, whereas *R*_iα_ are the coordinates of atom *i* and *τ* is an arbitrary temporal origin. The quantity inside the angular brackets is the square of the displacement that the molecule undergoes during the time interval *t*. The sum of all the molecules divided by the number of molecules gives the mean value called the MSD as a function of time *t*. Hence, the diffusion coefficient (*D*) can be obtained from the slope of the MSD curve using the Einstein relation,^37^ as follows:2
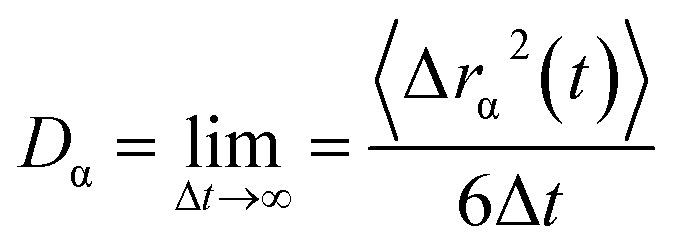


The atom's path is almost straight until it collides with its neighbors. If the system is solid (frozen), the MSD will be saturated and the kinetic energy will not be sufficient to achieve diffusion behavior. Likewise, if the system is not frozen (*e.g.* liquid), MSD increases linearly over time. Fig. S4† displays the calculated MSD of the Sc_2_Te_3_ system at temperatures above 673 K. The figure exhibits nearly linear behavior, implying that the mobility of atoms in the liquid increases gradually with increasing temperature. The MSD of individual atoms in the Sc_2_Te_3_ system shows that diffusion of Sc atoms is slightly greater than that of Te atoms, indicating that the mobility of Sc atoms is slightly faster than that of Te atoms. This may be due to the large difference in the atomic mass of Sc (44.96 g mol^−1^) and Te (127.6 g mol^−1^). The diffusivity of the system may depend on several factors, so the difference in atomic mass could not be the only reason allowing Sc atoms to diffuse faster than Te atoms in the Sc_2_Te_3_ system. For instance, the mobility of Te atoms in the Sb_2_Te_3_ system is slightly faster than that of Sb atoms.^14^ On the other hand, Sb atoms diffuse faster than Te atoms in the Ge–Sb–Te system.^38^ Likewise, the diffusivity of Sc is the smallest among the elements in the Sc-rich Sc–Sb–Te system.^12^

**Fig. 5:**
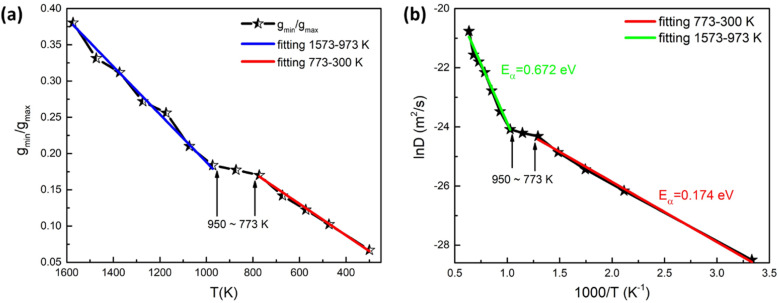
(a) The calculated Wendt–Abraham parameter of the Sc_2_Te_3_ system as a function of temperature. (b) The diffusion coefficient (*D*) of the Sc_2_Te_3_ system at different temperatures. The blue and green solid lines are the linear fitting results from 1573 to 973 K, while the red solid lines are the linear fitting results from 773 to 300 K.

The glass transition temperature is an important parameter used to estimate crystallization kinetics, such as fragility, viscosity, and crystal growth, in the chalcogenide PCMs.^39,40^ The WA parameters^41^ are used to determine the liquid-to-glass transition temperature of the Sc_2_Te_3_ system. The WA parameter is defined as *g*_min_/*g*_max_, where *g*_min_ and *g*_max_ correspond to the first minimum and maximum values of the PCF, respectively. Accordingly, the WA parameter of the Sc_2_Te_3_ system from 1573 K to 300 K is presented in Fig. 5(a). During the quenching process, the WA parameters are divided into two parts for linear fitting: the 1573–950 K part is marked by a blue solid line, and the 773–300 K part is marked by a red solid line. It is worth noting that the slopes of the two straight lines are quite different, indicating that the liquid-to-glass transition temperature is more likely to be between 773 K and 950 K.

Furthermore, the diffusion coefficient of Sc_2_Te_3_ as a function of temperature is also calculated. The diffusion coefficient (*D*) is usually described by the Arrhenius equation:^42^3
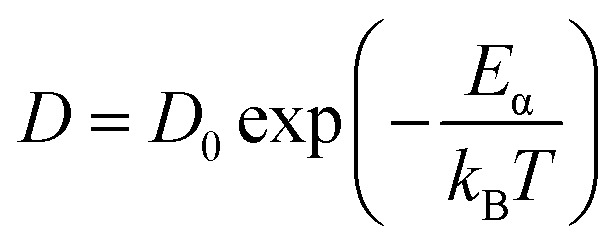
where *D*_0_ denotes the pre-exponential factor and *E*_α_ is the activation energy. Fig. 5(b) shows the relationship between ln(*D*) and 1/*T*. The calculated results are linearly fitted. The activation energy between 1573 K and 950 K (blue solid line) is 0.672 eV. As the temperature continues to decrease, the activation energy becomes 0.174 eV from 773 K to 300 K (red solid line). The sharp change in activation energy most likely shows a liquid-to-glass transition, which is consistent with the analysis of the WA parameter. However, the numerical simulations used to determine the glass transition in the PCMs are often ambiguous and controversial.^40^ It should be noted that the glass transition in the PCM is usually shown by experiments, and is difficult to accurately capture in our AIMD simulation. Hence, a large-scale *ab initio* simulation with more equilibration time would be required to accurately estimate the diffusivity based on the slope of the MSD curve.

### 3.3. Electronic properties

**Fig. 6:**
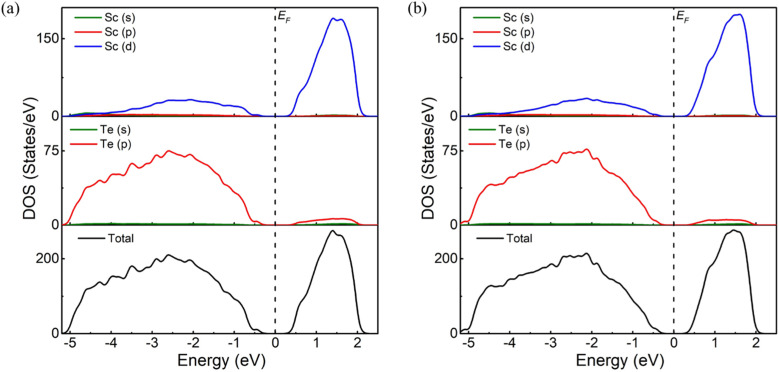
The total and partial density of states (DOSs). (a) DOSs of amorphous Sc_2_Te_3_ at 300 K and (b) DOSs of supercooled Sc_2_Te_3_ at 773K, using the mBJLDA functional. The dashed vertical line is the Fermi level (*E*_F_), corresponding to the zero point energy.

**Fig. 7:**
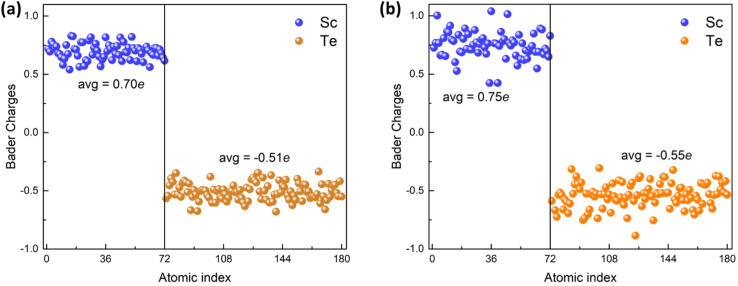
Bader charge distributions for an 180-atom Sc_2_Te_3_ system. (a) Bader charges of amorphous Sc_2_Te_3_ at 300 K and (b) Bader charges of supercooled Sc_2_Te_3_ at 773 K. Blue and orange dots represent 72 Sb and 120 Te atoms. The average values are given for both atomic species.

The electronic DOSs are calculated based on a new exchange and correlation potential called the mBJLDA functional, which can produce more accurate band gap energy than the PBE functional. Since small differences in the lattice parameters can cause significant differences in the value of the gap,^43^ the lattice parameters used for the DOS calculation are consistent with previous theoretical work.^13^ In this work, snapshots of the last trajectory are taken at 300 K and 773 K for electronic DOS calculations. The calculated total and partial DOSs are displayed in Fig. 6. The mBJLDA calculations show a widening of the energy gap, almost twice that obtained using the PBE functional.^13^ The DOS spectrum from the Fermi-level (*E*_F_) to −5 eV bonding energy constitutes the valence band region, and the region from *E*_F_ to 2 eV includes the conduction band region. In the entire energy range, the electronic DOS of the Sc_2_Te_3_ is mainly in the Sc_d state and the Te_p state. The lower valence band originates from the Te_5p states, while the Sc_3d states of Sc_2_Te_3_ play a leading role in the upper valence band. The contribution of s states is insignificant in the total DOS. The energy gaps between the first onset of the electron density from the left side (valence band) and the right side (conduction band) of the *E*_F_ are ∼−0.26 eV and ∼+0.26 eV. Note that all three crystalline phases of Sc_2_Te_3_ are metallic (Fig. S5†). This suggests that the electronic properties of the system change with temperature, manifesting as a metal-to-semiconductor transition. The Bader charge^44–46^ can also provide more electronic information based on the charge density. Fig. 7 shows the Bader charge distributions of Sc_2_Te_3_ at 300 K and 773 K. A positive value represents an electronic loss, and a negative value represents an electronic gain. There is a strong charge transfer from Sc to Te atoms. On average, each Sc atom donates around +0.7*e*, while the Te atom gains around −0.5*e*. This value is slightly lower than the charge value computed using the Löwdin method.^13^ Nevertheless, both methods show that substantial charge transfer occurs in the system, which favors the Sc-doped Sb_2_T_3_ phase-change material. For instance, the small charge transfer between Y and Te atoms in Y-doped Sb_2_Te_3_ (ref. 47) leads to the formation of non-octahedral bonds, which limits efficiency.

## 4. Conclusions

Using AIMD simulations, we have generated the liquid and glassy states of the Sc_2_T_3_ compound. The local structures and dynamic and electronic properties at different temperatures are investigated. The PCF analysis shows a strong interaction between Sc and Te atoms. The average CNs of Sc and Te atoms are closer to the ideal octahedral and tetrahedral coordination. The main peak of the Sc_2_Te_3_ ADF is the characteristic of the defective octahedral environment, indicating the presence of octahedral clusters in the system. The octahedral structure became more ordered as the temperature decreased. Ring statistics show that even-membered rings are dominant in Sc_2_Te_3_, similar to their crystalline counterparts. ABAB squares are the main motifs that can act as crystalline precursors, which is a key parameter for rapid incubation.

Based on the dynamic property that changes with temperature, it can be deduced that the liquid-to-glass transition temperature of Sc_2_Te_3_ is about 773 K to 950 K. The effect of temperature changes the electronic properties of the system, manifesting as a metal-to-semiconductor transition. The band gap obtained using the mBJLDA functional is twice the value obtained using the PBE functional. According to Bader charge analysis, there is a charge transfer from Sc to Te atoms. As a result, their net charges are around +0.7*e* and −0.5*e*, respectively. Our results highlight the effect of temperature on the structural, dynamic, and electronic properties of the Sc_2_Te_3_ system. Moreover, our results shed more light on the structural transition from liquid to amorphous states at the atomic level. We hope that this work could stimulate more theoretical work on the development of cache-type phase-change memory and stimulate further research interest in the field of PCM.

## Conflicts of interest

There are no conflicts to declare.

## Supplementary Material

RA-012-D2RA05720D-s001
