# Supplementary material for: Effect of temperature on structural, dynamical, and electronic properties of Sc2Te3 from first-principles calculations
Source: RSC Adv. 2022 Nov 15;12(51):32796–802. doi: 10.1039/d2ra05720d (PMC9665046; doi:10.1039/d2ra05720d)
Supplement: RA-012-D2RA05720D-s001 [file RA-012-D2RA05720D-s001.pdf]

Electronic Supplementary Material (ESI) for RSC Advances.
This journal is © The Royal Society of Chemistry 2022

Supporting Information

Effect of Temperature on Structural, Dynamical, and Electronic Properties of Sc_2Te_3 from First-Principles Calculations.

Getasew Mulualem Zewdie,*,† Tekalign Terfa Debela,# and Georgies Alene Asres¥

†College of mechanical and industrial engineering, Institute of Technology, Dire-Dawa University, Dire-Dawa, Ethiopia. E-mail: getasewmzewdie@hotmail.com

#Institute for Application of Advanced Materials, Jeonju University, Chonju, Chonbuk 55069, Republic of Korea

¥ Center for Materials Engineering, Addis Ababa Institute of Technology, School of Multi-disciplinary Engineering, Addis Ababa, 1000, Ethiopia.

* **Correspondence** and requests for materials should be addressed to G.M.Z.

Table S1 Average coordination numbers of different atomic pairs in the Sc_2Te_3 system at 300 and 773 K.

		With Sc	With Te	Total
**300 K**	**Sc**	0.003	5.634	5.637
	**Te**	3.756	0.003	3.759
**773 K**	**Sc**	0.029	5.346	5.375
	**Te**	3.564	0.026	3.590

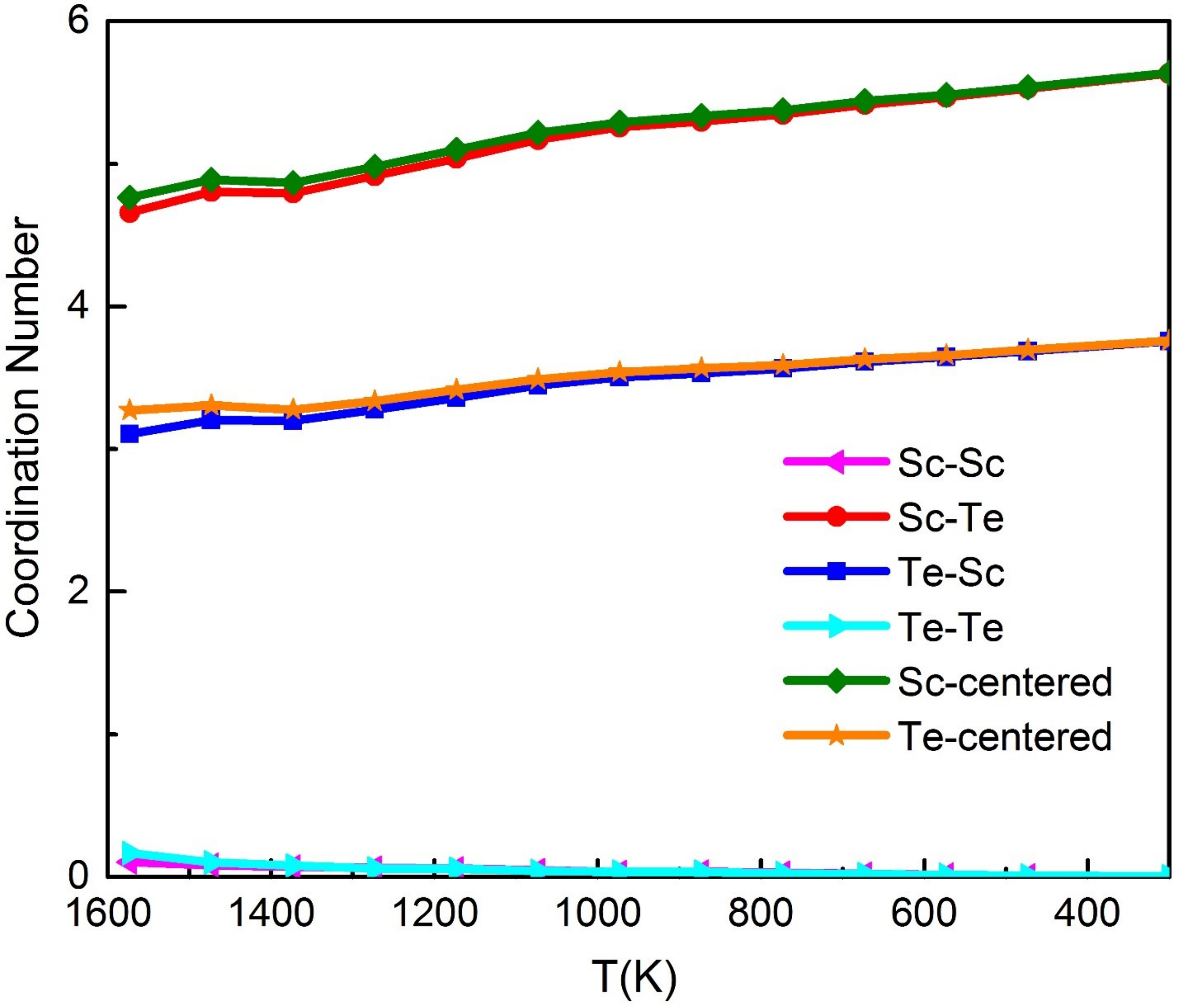

Figure S1 The A-B partial coordination number of the Sc_2Te_3 system as a function of temperatures. Here, A is the central atom and B is the coordination atom. The green and orange solid lines labeled as Sc-centered and Te-centered represent the total CNs of the Sc and Te atoms, respectively. The pink, red, blue, and cyan solid lines are labeled as Sc-Sc, Sc-Te, Te-Sc, and Te-Te partial CNs, respectively. A uniform cutoff distance of 3.3 Å is used.

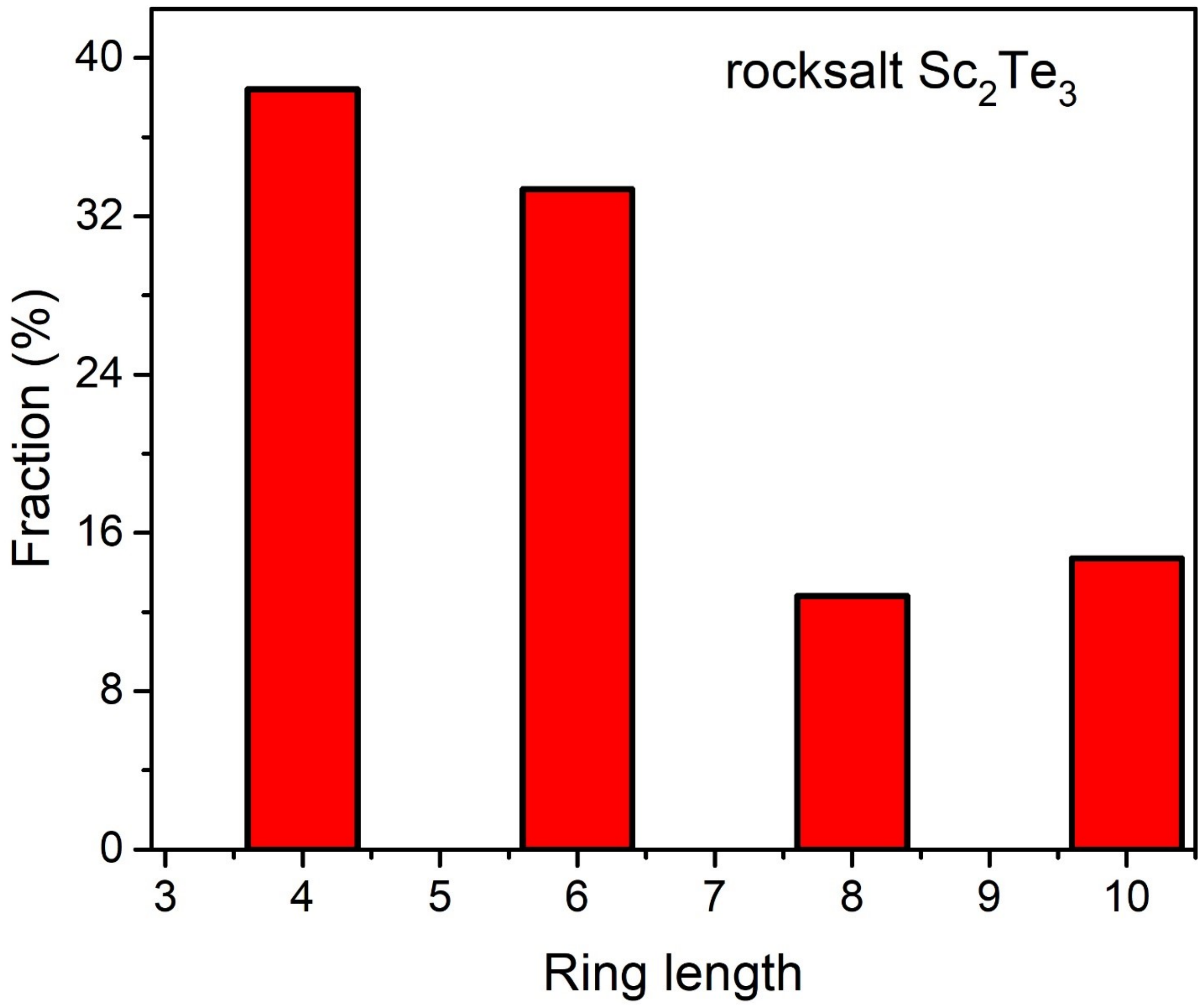

Figure S2 The primitive ring distributions in the cubic rocksalt Sc_2Te_3 structure.

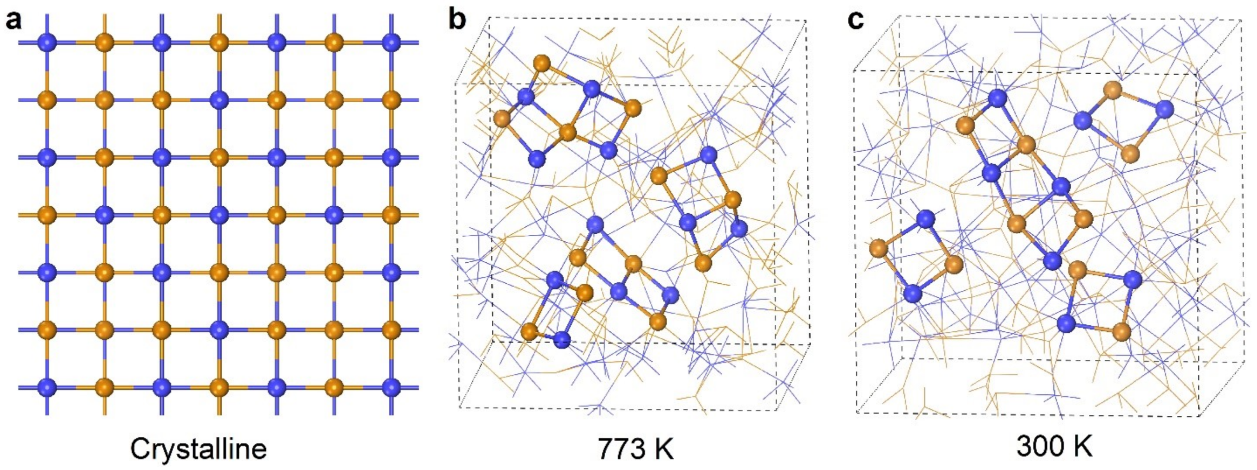

Figure S3 (a) The rocksalt-like crystal structures of Sc_2Te_3 with 1/3 vacancies on the cationic sub-lattice. Four-membered rings as the defining structural motifs are displayed. (b)-(c) The snapshots of typical four-membered primitive rings (ABAB squares) in the Sc_2Te_3 system at 773 K and 300 K, respectively, resembling the crystalline phase. The Sc and Te atoms are rendered in blue and orange balls, respectively.

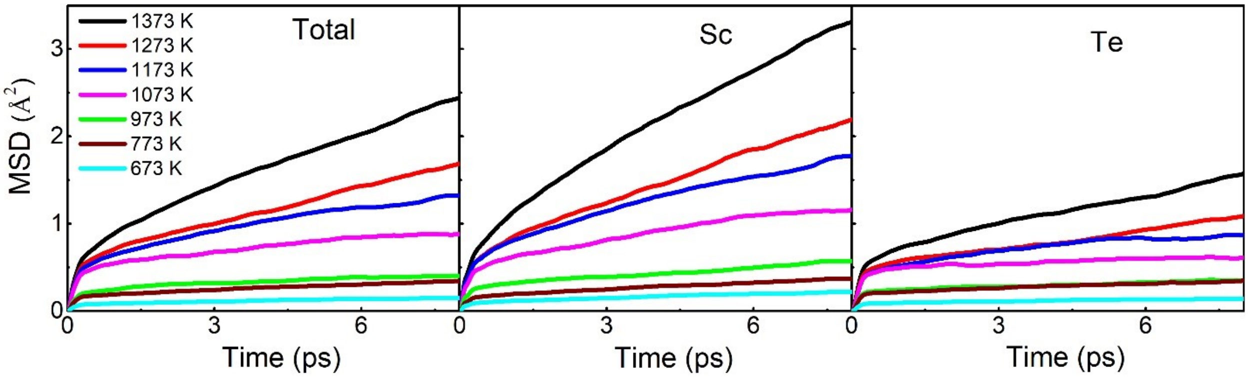

Figure S4 The Mean-squared displacement (MSD) of the Sc_2Te_3 system as a function of time at different temperatures.

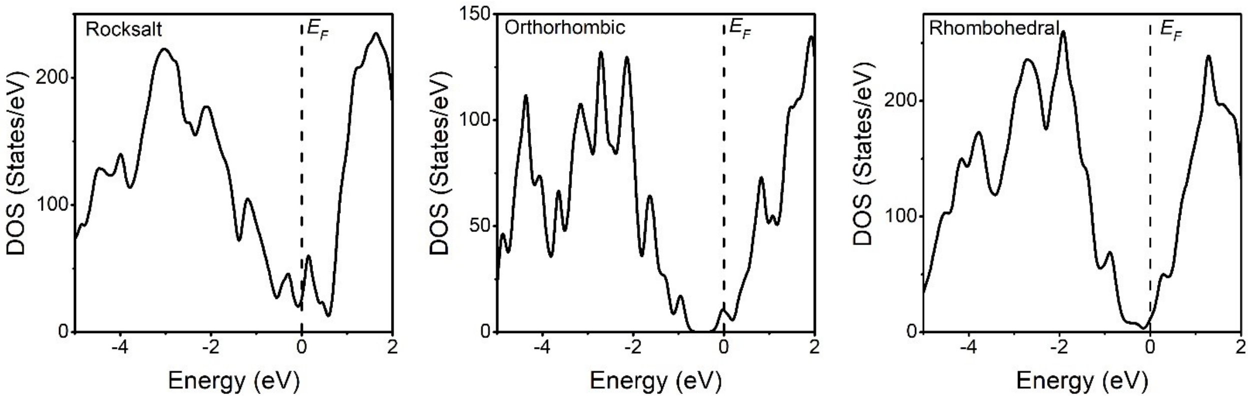

Figure S5 The total density of states (DOS) of crystalline phases of Sc_2Te_3 using the mBJLDA functional. (a)-(c) The total DOSs of the rocksalt, orthorhombic, and rhombohedral phases of the Sc_2Te_3 structure, respectively. The dashed vertical line is the Fermi level (E_F), corresponding to the zero point energy.
